# Evaluation of Disaster Medicine Preparedness among Healthcare Profession Students: A Cross-Sectional Study in Pakistan

**DOI:** 10.3390/ijerph17062027

**Published:** 2020-03-19

**Authors:** Ali Hassan Gillani, Mohamed Izham Mohamed Ibrahim, Jamshaid Akbar, Yu Fang

**Affiliations:** 1 Department of Pharmacy Administration and Clinical Pharmacy, School of Pharmacy, Xi’an Jiaotong University, Xi’an 710061, China; hassangillaniali@yahoo.com (A.H.G.); yufang@mail.xjtu.edu.cn (Y.F.); 2 Center for Drug Safety and Policy Research, Xian Jiaotong University, Xi’an 710061, China; 3 Shaanxi Centre for Health Reform and Development Research, Xian Jiaotong University, Xi’an 710061, China; 4 Department of Clinical and Pharmacy Practice, College of Pharmacy, QU Health, Qatar University, Doha P.O. Box 2713, Qatar; 5 Department of Pharmaceutical Sciences, The Superior College, Lahore 75500, Pakistan; dr.jamshaidsheikh@gmail.com

**Keywords:** natural disaster, man-made disaster, healthcare professionals, university students, low- and middle-income countries, curriculum development

## Abstract

*Background*: Disasters are devastating incidents, especially when occurring suddenly and causing damage, great loss of life, or suffering. Disasters can affect health and the social and economic development of a nation. The article analyzes the knowledge (K), attitude (A), and readiness to practice (rP) of healthcare professional students in universities in Pakistan. *Methods*: We carried out a cross-sectional study using a pretested and validated self-administered disaster medicine and preparedness questionnaire. The study recruited 310 students. Responses were scored and categorized as high (75th quartile), moderate (75–25th quartiles), and low (25th quartile). Independent *t*-test, one-way ANOVA, Pearson correlation, and regression analyses were performed at an alpha level of 0.05. *Results*: The study found that most of the students had moderate knowledge, attitude, readiness to practice, and total KArP scores. All K, A, and rP scores were significantly correlated with overall KArP scores. Knowledge and attitude factors were significant predictors of readiness to practice. *Conclusions*: We strongly believe that educators and health policymakers should build a strong curriculum in disaster medicine management and preparedness to prepare competent future healthcare professionals for the nation.

## 1. Introduction

Incidents are situations that may lead to disruptions, losses, emergencies, or crises. Disasters are devastating incidents, especially when occurring suddenly and causing damage, great loss of life, or suffering [1]. Disasters can affect the development of the healthcare sector. Attaining high standards of health and wellbeing is among one of the basic human rights documented in various regional, national, and international documents. Ensuring this right means easy access to essential medicines to treat chronic and communicable (e.g., water-borne or vector-borne) diseases during disasters [2]. Therefore, the provision of medication and health should be ensured through the medication supply system, and for this, efficient procurement and proper supply management is necessary [2,3]. However, human-made (wars, terrorism, and embargoes) and natural disasters (fires, hurricanes, floods, and earthquakes) are always a threat to the proper attainment of supplies and medication and the appropriate functioning of the healthcare system [4,5]. Unfortunately, disasters can occur at any place and at any time, resulting in healthcare system malfunction and impairment of the country’s economy [6].

To mitigate the effect of disasters, communities should be well aware of “how to respond to a disaster”, and healthcare professionals must be well-trained in managing disasters [6,7]. Disaster preparedness is defined as the “measures taken to prepare for and reduce the effects of disasters. That is, to predict and, where possible, prevent disasters, mitigate their impact on vulnerable populations, and respond to and effectively cope with their consequences” [8]. Therefore, it is affirmed that disaster management should be considered in the organizational context, with an emphasis on improving the knowledge (K), attitude (A), and practice (rP) of both the general public and healthcare professionals [7,8,9]. Therefore, the KArP of healthcare profession students should be evaluated and improved if required. Barriers to KArP should be better approached and improved at the institutional level by developing a strong curriculum, educational strategy, and training.

Pakistan is vulnerable to both human-made and natural disasters. Since its independence, the country has fought four wars with India—in 1947 (the Kashmir war), 1965, 1971, and 1999 (the Kargil war) [10]. Additionally, Pakistan was a pillar in the war against terrorism led by the United States, losing almost 50,000 civilians and 6000 members of security force staff. In 2013, 5379 Pakistanis were martyred in radical attacks, followed by 5496 in 2014, 3682 in 2015, 1830 in 2016, and 924 in 2017 [11,12]. Similarly, Pakistan is susceptible to natural disasters and has lost a considerable number of lives to floods, earthquakes, and landslides. In its history, more than 100,000 deaths have been rendered to earthquakes, with massive numbers of people injured and left homeless [13]. Similarly, floods also cause hundreds of deaths every year and a large number of injured; for example, in 2010, flooding led to the deaths of 1100 people and the displacement of 500,000 individuals [14]. Given such an inclination toward natural and human-made disasters, Pakistan should have a workforce that is prepared in disaster management. Training in disaster management would be of great assistance in increasing the available workforce to deal with calamities. To date, few studies have examined the viewpoint of prospective healthcare professionals on disaster medicine preparedness. Given the increasing number of disaster events which occur in the country, a study is crucial.

However, previously assessed levels of preparedness in relation to disaster management were found to be insufficient in many countries [15,16]. For example, a study carried out with rural center healthcare staff in Australia found that the level of preparedness for, knowledge of, and willingness to respond to calamities was low given insufficient knowledge, lack of training activities, and limited experience with disasters [15].

Similarly, studies among healthcare providers from developing nations such as Yemen, Saudi Arabia, China, Ethiopia, and Malaysia have demonstrated a knowledge and level of preparedness to handle disasters ranging from inadequate to somewhat satisfactory [16,17,18,19,20,21]. Also reported was that the unavailability of training activities was a major factor hindering health professionals’ knowledge regarding disaster preparedness in Yemen and Ethiopia [18,19].

Studies on healthcare students that considered the role of students as future healthcare providers demonstrated similar results. For instance, students from the European countries of Germany, the Netherlands, and Italy revealed low confidence and knowledge in disaster management [22,23,24]. Conversely, a study undertaken in the United States to assess the knowledge, attitudes, and confidence in practice levels of nursing, medicine, and dental students in the event of disasters demonstrated ample knowledge and understanding of the existing set of courses and a need for only slight changes [25].

Future healthcare experts, policymakers, educators, doctors, nurses, and pharmacists should be taught. In addition, they should appropriately trained in disaster management and preparedness because previous results make it evident that responses to disasters are inadequate [26]. Students in the health profession that belong to low- and middle-income countries (LMICs) are considered to have inadequate knowledge of this subject. Disaster management and preparedness courses would be of great assistance in strengthening the healthcare system’s workforce so that it is available to deal with calamities. To date, the number of available studies that evaluate the perspectives of future healthcare professionals on disaster medicine preparedness issues is limited. Therefore, in accordance with the importance of this issue, this study aims to assess the level of disaster medicine preparedness among healthcare students in Pakistan by assessing and evaluating their knowledge (K), attitude (A), and readiness to practice (rP) regarding disaster medicine and preparedness.

## 2. Materials and Methods

### 2.1. Study Design

A quantitative survey-based cross-sectional study was conducted among students of the Superior University College of Pharmacy (SUCP) in Lahore, Shaikh Khalifa bin Zayed Al Nahyan Medical and Dental College (SKZMDC) affiliated to the University of Health Sciences in Lahore, Nishtar Dental College (NDC) in Multan, and Quaid-E-Azam Medical College (QAMC) in Bahawalpur. The former is a private college of pharmacy, and the latter three are government-owned medical colleges. Data were collected between May and October 2019.

### 2.2. Ethical Considerations

Before its commencement, this study was approved by the institutional review committee of Superior College of Pharmacy (Ethical approval number: SUP-2019-DM/1). In addition, consent was obtained from each institutional managing authority. Verbal consent was obtained from all of the participants after the objectives, importance, and benefits of the research and the voluntary nature of participation were discussed. Participants were assured that all of the data gathered would be handled with full confidentiality and would be used solely for research purposes. They were made aware that they can withdraw anytime. No names were identified on the questionnaires. The researchers only used codes on the forms and in analysis; thus, the data cannot be linked to the participants.

### 2.3. Study Participants and Sample Size

Pharmacy students from the SUCP (*n* = 250) and medical/dental students from SKZMDC (*n* = 300), NDC (*n* = 355) and QAMC (*n* = 352) were captivated in the study. From a total of almost 1257 students, a sample of 303 students was required. The sample size was estimated using Raosoft [27] based on a 5% margin of error and considering a 95% confidence level and a response rate of 80%. Choosing different colleges, students were approached on the basis of convenience. Undergraduates from the SUPC, SKZMDC, NDC, and QAMC were included in the research with no limitations on gender, age, or study level/academic year. Students from the SUCP participated in pilot testing; all first-year students and postgraduate levels were excluded from the study.

### 2.4. Tool Development

Questionnaire development was based on review of literature [9,18,22]. A pre-validated questionnaire was already being used in a study in Qatar [26]. Study tools involved the three primary spheres of knowledge, attitude, and readiness to practice (KArP), with participants’ demographics in a separate domain (see Supplementary File S1). Section 1 was a knowledge section, including 22 close-ended binary questions (yes/no as an answer) with a range of 0–22 points. To categorize points, cut-offs were set as follows: fewer than 7 points were considered low scores (25th quartile), scores ranging from 7–12 points were taken as moderate (26–75th quartiles), and more than 12 points were considered high (>75th quartile). Section 2, the attitude section, had 16 Likert scale questions (strongly agree, agree, neither agree nor disagree, disagree, strongly disagree), with a minimum of 16 and a maximum of 80 possible points. Scores of less than 42 points were considered low, scores of between 42 and 56 points were considered moderate (25–75th quartiles), and scores of more than 56 points were considered high. The readiness to practice section consisted of 11 Likert scale questions (strongly agree (1), agree (2), neither agree nor disagree (3), disagree (4), strongly disagree (5), and not applicable (6)) that could reach a total of 55 points. Scores below 31 points, 31–38 points, and higher than 38 points were considered low (25th quartile), moderate (higher than the 25th quartile and lower than the 75th), and high (75th quartile), respectively.

Pilot testing, after the development of the tool, was conducted with 10 SUCP students. Minute modifications, including deletions or additions, were subsequently made in response to student feedback, with the content of all of the items remaining unchanged. We re-ran the validity measures of the tool, which indicated that the Cronbach’s alpha values for K, A, rP, and total KArP were 0.793, 0.840, 0.705, and 0.888, respectively.

### 2.5. Data Collection

A self-administered survey was conducted to collect data from the students of the selected colleges. A representative of each individual college/university was appointed to approach the respondents to increase participation.

### 2.6. Data Analysis

The data were analyzed using SPSS V-26 (IBM Corp. Released 2018. IBM SPSS Statistics for Windows, Version 26.0. Armonk, NY, USA). The normality of the results was checked using the Kolmogorov–Smirnov test. Descriptive analysis, frequency (%) for noncontinuous variables, and/or mean (±SD) or median (IQR) for continuous variables were used. Because the data were not normally distributed, nonparametric tests (i.e., Kruskal–Wallis and Mann–Whitney) were used. The correlation among the three parameters (K, A, and rP) was examined using Pearson’s correlation test. Linear regression was performed to predict the readiness to practice (independent variable) from knowledge and attitude (independent variables). All tests were carried out at an a priori alpha level of 0.05.

## 3. Results

Table 1 shows the characteristics of the students. Most of the students were female (*n* = 174, 56.9%), majoring in medicine (*n* = 204, 65.8%), and in their senior years, that is, fourth and fifth years (*n* = 246, 79.4%).

Table 2 illustrates the association between the characteristics of students using the K, A, rP, and KArP scores. The age factor was shown to be significantly negatively correlated with the K, A, rP, and overall KArP scores (*p* < 0.001). Only knowledge was moderately correlated, whereas other factors were weakly correlated. Gender was proven to be not significantly related to the K, A, rP, and KArP scores. Further analyses on the differences in degree major, university, and academic year with K, A, rP, and KArP scores indicated significant differences (*p* < 0.05).

Table 3 indicates the relationship between the demographic and academic profiles with different K, A, rP and KArP categories. K, A, rP, and KArP scores clearly indicated that most of the students had moderate knowledge, attitude, readiness to practice, and total KArP levels. Further analyses revealed that gender, degree major, university, and academic year showed significant differences (*p* < 0.001) with respect to knowledge; degree major, university, and academic year showed significant differences (*p* < 0.05) with respect to attitude; gender and academic year showed significant differences (*p* < 0.05) with respect to readiness to practice; and degree major, university, and academic year showed significant differences (*p* < 0.001) with respect to total KArP.

The correlation between the K, A, rP, and KArP scores was carried out before extending to the regression analysis. The findings indicated that K–A, K–P, and A–P were significantly associated (*p* < 0.001). In addition, all K, A, and rP scores were strongly significantly correlated with the KArP score Table 4.

### Regression Analysis

When readiness to practice was predicted, knowledge (Beta = 0.345, *p* < 0.01) and attitude (Beta = 0.192, *p* < 0.01) were found to be significant predictors. The overall model fit was R^2^ = 0.732. The total variation in the dependent variable of readiness to practice can be explained by the independent variables of knowledge and attitude, at 73.2%. ANOVA revealed that the regression equation fit very well (*p* < 0.01) with the data (i.e., predicts the readiness to practice). The regression equation can be presented as Equation (1):

rP = 0.192 K + 0.345 A + 11.113(1)

## 4. Discussion

In this study, we evaluated the knowledge, attitude, and readiness to practice among healthcare profession students in several universities in Pakistan regarding disaster medicine management and preparedness. Our findings indicated that, in general, the scores for K, A, rP, and KArP of the students were moderate. These scores reconcile the aspects of the literature that show evidence of lack of knowledge, positive attitude, and level of readiness to practice among healthcare profession students, especially in LMICs. Thus, there is a need for collective efforts from various stakeholders to overcome these limitations.

In the past, the world has observed a significant increase in the intensity and frequency of disasters, both natural and human-made. Undeniably, disaster risk reduction is a vital element of social and economic progress, especially to ensure the future sustainability of development. Countries—LMICs in particular—must increase their efforts to prepare healthcare professionals for disaster management and reduction. The United Nations Office for Disaster Risk Reduction (UNISDR) reported on the economic losses to low- and lower-middle-income countries caused by disasters [28]. Approximately 1.3 million people were killed by disasters from 1998–2017 [28]. Additionally, 4.4 billion people were injured, made homeless, or in need of emergency assistance because of disasters. In 2016, the Insurance Information Institute [29] reported 327 disaster events, of which 136 (42%) were human-made. According to ReliefWeb 2019 [30] the World Bank reported that Pakistan is among the most vulnerable countries in South Asia, and has suffered an estimated US$ 18 billion in damages and losses. Pakistanis are exposed to various types of disasters, such as those of a geophysical, hydrological, meteorological, climatological, and biological nature, as well as war and terrorism [31]. According to GermanWatch, the vulnerability index of 2019 ranked Pakistan eighth among the 10 countries most affected by extreme weather events between 1997 and 2016 [32].

Disaster medicine involves the care of victims of natural and human-made disasters, is concerned with the health, medical, and emotional aspects of disasters, and includes disaster management. Healthcare professionals need to be competent and willing to respond to disasters and need to be involved in the areas of preparedness, recovery, and mitigation [33]. Preparedness is one of the four phases of the disaster medicine cycle. The other phases include planning and mitigation, response, and recovery. For healthcare professionals to be competent and willing to respond to disasters, disaster medicine preparedness should be the central component of the curriculum for healthcare profession students.

Education can ensure the sustainable development process of the nation. Acquiring knowledge is regarded as an effective way to prevent disasters or to reduce its effects. According to Torani et al. disaster education is a functional, operational, and cost-effective risk management tool. Planning and designing comprehensive educational programs are necessary for people to face disasters [34]. The integration of disaster medicine management and preparedness in higher education institutions is crucial [34,35]. Education and training can and will increase the awareness regarding the causes, risks, and implications of disasters on financial, social, economic, environmental, and humanitarian aspects.

According to Hoffmann and Mutarrak [36], formal education is important in promoting disaster preparedness. Early education and training enhance levels of knowledge and preparedness by improving awareness and the perception of the risk of a disaster [37]. Overall, our study indicated that the knowledge levels related to disaster medicine management and the preparedness of Pakistani students were moderate. Factors that were significantly related to knowledge were student’s age, degree major, university, and academic year. Our findings are consistent with other studies. For example, Bajow et al. found that students’ knowledge levels before implementing an intervention were unsatisfactory; gender and years of academia were not significant factors [38]. One study in China indicated that there were low levels of knowledge of disaster psychology and disaster administration among medical students [39].

Patel et al. raised concerns about a significant lack of emphasis on disaster preparedness in healthcare curricula and the demand for wider inclusion of disaster training to improve the willingness to work among healthcare students [40]. Kaiser and colleagues highlighted the relatively limited exposure by medical students to disaster preparedness topics. Furthermore, according to their study, future physicians’ willingness to respond, education and training in disaster medicine, and public health preparedness courses offered in US medical schools were inadequate [41].

What topics should be included in the courses, and what are the appropriate methods for delivering these courses? Mortelmans et al. studied the outcomes of introducing disaster medicine courses. Among the aspects covered were chemical, biological, radiological, disease pandemic, and nuclear training. Their study found that military students scored higher on knowledge and capability and were better prepared for disaster situations than their civilian counterparts, that is, medical science students [42]. Ingrassia and coworkers studied the disaster management educational and training initiatives at the postgraduate level in EU countries and found that face-to-face education was the most common teaching method used. The contents were multi- and cross-disciplinary, and a competency-based approach to curriculum content was present [43]. Bajow introduced a new undergraduate community-based disaster medicine course [38]. To promote higher cognitive engagement, the curriculum introduces core principles in emergency medicine, public health, and disaster management. Various approaches were used, including lectures, workshops, simulations, group discussions, case studies, and role-playing. Five major domains were presented in this curriculum: (1) general concepts of disaster medicine; (2) disaster risk reduction; (3) disaster and mass casualty incident management; (4) principles of community awareness; and (5) training sessions for community education [38]. Kaji et al. developed and evaluated a 2-week disaster elective. The elective consisted of 16 lectures, field visits, and experiential activities, including observation of a statewide disaster drill [44]. The course by Cole et al. used a variety of teaching modalities, including lectures, videos, and tabletop and hands-on simulation exercises. The subject matter included biological and chemical terrorism, disaster management, mechanisms of injury, and psychiatry [45]. Pfenninger and coworkers developed a course that consisted of 14 modules and that was composed of 2-h units. The concepts of disaster medicine included response, medical assistance, law, command, coordination, and communication, and the introduction of mass casualty management [46]. The courses were based on their review of various worldwide hospital preparedness plans and disaster assistance experiences. In addition, the teaching and training modules included aspects of life-saving emergencies, management of explosives, and war-related radiological/nuclear, chemical, and biological incidents (emphasizing infectious diseases and terrorist attacks), as well as an evacuation exercise and a mass casualty triage. Decontamination procedures were demonstrated at a nuclear power plant or the local fire department, and participants were involved in personal decontamination practices. They practice resuscitation procedures using mannequins. The course included an interactive review of professional ethics, stress disorders, psychosocial interventions, and quality improvement efforts. Montana and colleagues shared their experiences in developing an elective course on the pharmacist’s role in disaster management for third-year pharmacy students and evaluated its effects on students’ knowledge and perceptions on introducing this course into the curriculum. They concluded that the potential exists to increase the number of pharmacists prepared to respond to disasters. The course also expanded students’ understanding of the pharmacist’s role and inspired them to be involved in emergency preparedness [47].

All of the curricula previously mentioned offer disaster medicine management and preparedness education to college educators during a reasonable period in an interdisciplinary multiexperiential course. Several strategies are also used to teach and train students.

When we evaluated students’ attitudes toward disaster medicine management and preparedness, their levels were moderate. Degree major, university, and academic year are significantly related to attitude. Kim et al. studied the outcomes of a discipline-specific online didactic course relative to an interdisciplinary course. Participants were senior students from the fields of allied health, medicine, nursing, and pharmacy. The findings indicated that students showed positive changes in both knowledge and attitudes in a critical event response course that included interprofessional instruction and collaboration [48]. In disaster medicine management, interprofessional learning is recognized as a necessary component. A study among US medical students found that they were willing to respond to disaster scenarios. The authors recommended that medical students should be prepared with sufficient knowledge, skills, and direction [41]. Another US study by Sauser and coworkers among allopathic medical students assessed their levels of preparedness and willingness to perform medical procedures in the event of a disaster. They discovered that the students have skills that could be beneficial when responding to a disaster. [49]. Ragazzoni et al. carried out a study in Italy and found that more than four-fifths of medical students had a positive attitude toward disaster preparedness. These students reportedly welcomed the introduction of a course on disaster medicine in their core curriculum, and the majority of the respondents considered knowledge of disaster medicine to be important for their future professions. [23]. Although German students were highly motivated, the majority were not well educated regarding disaster medicine [22]. A study in Belgium indicated that the respondents wanted to increase their knowledge in this area and welcomed the introduction of specific courses into the standard medical curriculum [42].

Our study illustrated that students’ levels of readiness to practice were moderate. Moreover, student gender and academic year showed significant differences with respect to readiness to practice. A study in Turkey among nursing students found that respondents seemed more likely to participate in disaster preparedness and recommended including disaster management skills and mass casualty care into the undergraduate curricula [50]. Kaiser et al. found that more than four-fifths of medical students reported a willingness to respond to a natural disaster, pandemic influenza, and a radiological event [41]. An analysis of the total KArP showed that degree major, university, and academic year of the students were significantly associated with the overall KArP score. The findings indicated that the relationship between K-A, K-rP, and A-rP were significant. In addition, all K, A, and rP scores were strongly significantly correlated with the KArP score.

### 4.1. Limitations and Strengths of the Study

This study has a few limitations. One limitation is that the results are expressed in a Pakistani environmental, cultural, and educational context, which may differ from that of other countries. Therefore, the findings might not be suitable for generalization to other LMICs. According to Brown et al., respondent attitudes, perceptions, and behavior toward disasters can be affected by exposure to and experience with such events. However, this study did not measure respondents’ experience and exposure [51]. This study only focused on a few geographical areas of Pakistan. Because Pakistan has a larger area and more critical geographical areas such as Kashmir, the study should be expanded to other regions. However, because the study relied on a survey, self-reported data and self-perception might have caused biases. In contrast, this study contributes another important perspective from future healthcare professional students in LMICs.

### 4.2. Recommendations and Implications

To advance the disaster prevention management and given a lack of information on the KArP among healthcare profession students in Pakistan, the results of this study can be used by colleges, research centers, policymakers, and health and disaster authorities to improve educational aspects in health colleges. Education is one of the fundamental factors of development. Countries, particularly those in resource-poor settings such as Pakistan, can achieve sustainable social, economic, and environmental developments through substantial investments in human capital. Education can create people’s creativity and improve their abilities. The recommendation is that authorities and health educators should prioritize according to different categories of education content, and more training can be considered for more important areas. Health colleges should review their curricula, assessment methods, and study plans. Disaster medicine management and preparedness are about health professionals working together. Hence, interprofessional education is crucial. Healthcare profession students should learn together during their professional training. The intention is to cultivate collaborative healthcare training. Colleges should also hire competent faculty members who can teach and train students in disaster management. Health educators and college administrations need to decide to include the course as either an elective (i.e., student can choose) or a core course (i.e., mandatory for all students).

Research can be designed to test the effectiveness of the courses (e.g., learnability, behavior change, skills on the job) and teaching strategies (e.g., didactic, group work, seminar, assignment, technology integration) using study designs such as a pre-post design or a mixed-method approach. Health colleges can also invite experts from various relevant organizations (the United Nations and its organizations, Health Care in Danger project, the International Federation of Red Cross and Red Crescent Societies, the International Committee of the Red Cross, or local organizations) to teach and share their experiences with students. Researchers can also assess students’ satisfaction and acceptance levels. Given the increasing global frequency of disasters [52], future healthcare professionals must be educated and trained to effectively respond to disaster situations when they become healthcare professionals. Academic institutions can contribute leadership and resources to societal disaster preparedness [53]. Several studies strongly recommended that continuous and consistent disaster knowledge, skills, and preparedness content be included in college curricula [50,51,52,53,54,55,56].

## 5. Conclusions

Disasters can affect the development of several aspects in the healthcare sector, and there is concern regarding public access to medicines. Healthcare professionals must be well trained in managing disasters. Students of the healthcare profession are the country’s future healthcare professionals. Healthcare profession students’ knowledge, attitude, and readiness to practice should be evaluated and improved if required. Given the lack of research in Pakistan, we decided to conduct a study among health profession students.

In summary, our study concluded that the levels of knowledge, attitude, and readiness to practice of health profession students in Pakistan with respect to disaster medicine management and preparedness were moderate. All K, A, and rP scores were strongly significantly correlated with the KArP score. Knowledge and attitude factors were significant predictors of readiness to practice. If initiatives are focused on improving knowledge and attitude, readiness to practice can be enhanced. Barriers to KArP should be better addressed and improved at the institutional level by developing a strong curriculum, educational strategy, and training.

## Figures and Tables

**Table 1:** Demographic and academic profile. SUCP: Superior University College of Pharmacy; NDC: Nishtar Dental College; QAMC: Quaid-E-Azam Medical College; SKZMDC: Shaikh Khalifa bin Zayed Al Nahyan Medical and Dental College.

Characteristics	Subitem	Frequency (*n*)	Percentage (%)
Age	Mean (SD) 23.6 (1.8)		
Gender	Female	174	56.9
	Male	136	43.1
Degree Major	Pharmacy	89	28.7
	Medicine	204	65.8
	Dental	16	5.2
University	SUCP	94	30.3
	NDC	96	31.0
	QAMC	82	26.5
	SKZMDC	38	12.3
Academic Year	Second	17	5.5
	Third	47	15.2
	Fourth	127	41.0
	Final	119	38.4

**Table 2:** Relationship between knowledge, attitude and readiness to practice scores with the respondents’ demographic and academic profiles.

Characteristics	Subitem	Knowledge (K) Score (*n* = 310)	*p*-Value	Attitude (A) Score (*n* = 310)	*p*-Value	Readiness to Practice (rP) Score (*n* = 310)	*p*-Value	Total KArP Score (*n* = 306)	*p*-Value
Age		r = −0.637	0.000 *	r = −0.153	0.007 *	r = −0.243	0.000 *	r = −0.354	0.000 *
		median		median		median		median	
Gender	Female	28.0	0.654 **	36.0	0.250 **	28.0	0.545 **	90.0	0.456 **
	Male	27.0		36.0		29.5		92.0	
Degree Major	Pharmacy	32.0	0.000 ***	39.0	0.002 ***	30.0	0.000 ***	101.0	0.000 ***
	Medicine	26.0		35.0		27.0		90.0	
	Dental	31.0		45.0		32.0		108.0	
University	SUCP	32.0	0.000 ***	37.5	0.045 ***	30.0	0.000 ***	99.0	0.000 ***
	NDC	26.0		36.0		27.0		89.0	
	QAMC	26.0		35.0		27.0		89.0	
	SKZMDC	34.0		35.0		30.0		104.0	
Academic Year	Second	34.0	0.000 ***	32.0	0.003 ***	26.0	0.000 ***	92.0	0.000 ***
	Third	31.0		36.0		30.0		96.0	
	Fourth	27.0		37.0		28.0		90.0	
	Final	26.0		33.0		27.0		89.0	

Note: * Spearman rho test; ** Mann–Whitney test; *** Kruskal–Wallis test. *p*-value in bold means it is significant.

**Table 3:** Demographic and academic profile associations with different K, A, rP, and KArP categories.

Characteristics	Subitem	Low Score	%	Moderate Score	%	High Score	%	*p*-Value *
Knowledge (*n* = 310)		46	14.8	180	58.1	84	27.1	-
Attitude (*n* = 310)		75	24.2	149	48.1	86	27.7	-
Readiness to Practice (*n* = 306)		34	11.0	194	62.6	78	25.2	-
Total KArP (*n* = 306)		67	21.6	162	52.3	77	24.8	-
**Association of demographics and academic profiles with knowledge categories**								
Gender	Female	34	19.5	83	47.7	57	32.8	0.000
	Male	12	8.8	97	71.3	27	19.9	
Degree Major	Pharmacy	6	6.7	31	34.8	52	58.4	0.000
	Medicine	40	19.6	139	68.1	25	12.3	
	Dental	0	0.0	9	56.3	7	43.8	
University	SUCP	6	6.4	33	35.1	55	58.5	0.000
	NDC	22	22.9	73	76.0	1	1.0	
	QAMC	18	22.0	58	70.7	6	7.3	
	SKZMDC	0	0.0	16	42.1	22	59.9	
Academic Year	2nd	1	5.9	7	41.2	9	52.9	0.000
	3rd	1	2.1	25	53.2	21	44.7	
	4th	14	11.0	76	59.8	37	29.1	
	Final	30	25.2	72	60.5	17	27.1	
**Association of demographics and academic profiles with attitude categories**								
Gender	Female	39	22.4	85	48.9	50	28.7	0.701
	Male	36	26.5	64	47.1	36	26.1	
Degree Major	Pharmacy	21	23.6	28	31.5	40	44.9	0.000
	Medicine	48	23.5	120	58.8	36	17.6	
	Dental	6	37.5	0	0.0	10	62.5	
University	SUCP	23	24.5	32	34	39	41.5	0.002
	NDC	23	24.0	57	59.4	16	16.7	
	QAMC	23	28.0	42	51.2	17	20.7	
	SKZMDC	6	15.8	18	47.4	14	36.8	
Academic Year	2nd	4	23.5	12	70.6	1	5.9	0.023
	3rd	7	14.9	23	48.9	17	36.2	
	4th	25	19.7	17	52.8	35	27.6	
	Final	39	32.8	47	39.5	33	27.7	
**Association of demographics and academic profiles with readiness to practice categories**								
Gender	Female	7	4.0	127	73.0	40	23.0	0.000
	Male	27	20.5	67	50.8	38	28.8	
Degree Major	Pharmacy	8	9.4	49	57.6	28	32.9	0.131
	Medicine	23	11.3	138	67.6	43	21.1	
	Dental	3	18.8	6	37.5	7	43.8	
University	SUCP	8	8.9	54	60.0	28	31.1	0.621
	NDC	11	11.5	60	62.5	25	26.0	
	QAMC	12	14.6	54	65.9	16	19.5	
	SKZMDC	3	7.9	26	68.4	9	23.7	
Academic Year	2nd	3	17.6	14	82.4	0	0.0	0.044
	3rd	3	6.4	30	63.8	14	29.8	
	4th	10	8.1	74	60.2	39	31.7	
	Final	18	15.1	76	63.9	25	21.0	
**Association of demographics and academic profiles with KArP categories**								
Gender	Female	38	21.8	87	50,0	49	28.2	0.350
	Male	29	22.0	75	56.8	28	21.2	
Degree Major	Pharmacy	10	11.8	33	38.8	42	49.4	0.000
	Medicine	51	25.0	128	62.7	25	12.3	
	Dental	6	37.5	0	0.0	10	62.5	
University	SUCP	10	11.1	39	43.3	41	45.6	0.000
	NDC	27	28.1	63	65.5	6	6.3	
	QAMC	24	29.3	49	59.8	9	11.0	
	SKZMDC	6	15.8	11	28.9	21	55.3	
Academic Year	2nd	4	23.5	10	58.8	3	17.6	0.005
	3rd	5	10.6	22	46.8	20	42.6	
	4th	26	21.1	60	48.8	37	48.8	
	Final	32	26.9	70	58.8	17	14.3	

Note: * Chi Square test; *p*-value in bold means it is significant

**Table 4:** Association between knowledge, attitude, and readiness to practice scores.

Characteristics	Statistical Values	Attitude	Readiness to Practice	KArP
Knowledge	r	0.458	0.504	0.704
	*p*-value	**0.000**	**0.000**	**0.000**
	*n*	310	306	306
Attitude	r	-	0.738	0.917
	*p*-value	-	**0.000**	**0.000**
	*n*	-	306	306
Readiness to Practice	r	-	-	0.862
	*p*-value	-	-	**0.000**
	*n*	-	-	306

Note: Spearman rho test; r = correlation coefficient; *n* = sample size. *p*-value in bold means it is significant.

## Supplementary Materials

The following are available online at https://www.mdpi.com/1660-4601/17/6/2027/s1, File S1: Survey Assessment of Disaster Medicines Preparedness and Readiness to Practice among Healthcare Profession University/Medical Students in Pakistan.
