# Supplementary material for: Evaluation of Disaster Medicine Preparedness among Healthcare Profession Students: A Cross-Sectional Study in Pakistan
Source: Int J Environ Res Public Health. 2020 Mar 19;17(6):2027. doi: 10.3390/ijerph17062027 (PMC7143317; doi:10.3390/ijerph17062027)
Supplement: Supplementary file 1 [file ijerph-17-02027-s001.pdf]

Supplementary File S1:

Survey Assessment of Disaster Medicines Preparedness and Readiness to Practice among Healthcare Profession University/Medical Students in Pakistan

In this survey you will be asked some questions to evaluate your knowledge, attitude, and readiness components in case of a **disaster affecting the medicine supplies**. The targeted population of this survey is the healthcare profession University/Medical students. Please read the questions very well and provide your answers accordingly. Please note that all the information you provide us is totally CONFIDENTIAL, and would be used only by the research team for the purposes of research.

Disaster management means "The organization and management of resources and responsibilities for dealing with all humanitarian aspects of emergencies, in particular preparedness, response and recovery in order to lessen the impact of disasters".

Disaster medicine is the area of medical specialization serving the dual areas of providing health care to disaster survivors and providing medically related disaster preparation, disaster planning, disaster response and disaster recovery leadership throughout the disaster life cycle.

Part I: Demographic information

Please answer the following questions.   Put a check ✓ mark.

Your gender	□ Female □Male
Your age (years)	
Your degree major	□Pharmacy □Medicine□ Dental □Laboratory □ Nursing
University name	
Your academic level (year)	

Part II: General

Please answer the following questions:

1) What is **the main thing** that comes to mind when you think about access to medicines?	
2) What is **the most important** thing related to medicines to consider during the disaster crisis?	
3) Many of the medicines used in Pakistan are original branded products. What is your opinion regarding generic substitution of prescribed medicines for cheaper	

alternatives (**the most important** concern)?	
4) What is **the best thing** about the health system in Pakistan?	
5) What is **the worst thing** about the health system in Pakistan?	

Part III (a): Knowledge Assessment

Please read the following statements and answer with a check mark (✓) for "Yes" or "No".

	Yes	No
1. I have previous exposure to this topic (*Disaster Medicines Preparedness*).	☐	☐
2. I have previous experience in dealing with disasters.	☐	☐
3. I think Pakistan is at risk of disasters (natural or human made).	☐	☐
4. Disasters come in many shapes and sizes.	☐	☐
5. Disaster medicine is the sole responsibility of Pharmacy organization.	☐	☐
6. I read journal articles related to medicines disaster preparedness.	☐	☐
7. I am aware of classes about disaster medicines preparedness and management that are offered for example at either my college, or community.	☐	☐
8. I find that the research literature on disaster medicines preparedness and management is easily accessible.	☐	☐
9. I find that the research literature on disaster medicines preparedness is understandable.	☐	☐
10. Finding relevant information about disaster medicines preparedness related to this country needs is an obstacle to my level of preparedness.	☐	☐

11. I know where to find relevant research or information related to disaster medicines preparedness and management to fill in gaps in my knowledge.	☐	☐
12. I know referral contacts in case of a disaster medicines situation (e.g. health department).	☐	☐
13. In case of a disaster medicines situation I think that there is sufficient support from local officials on the governance level.	☐	☐
14. I am aware of what the potential risks emergencies in this country are (e.g: natural disaster, embargo, terror, war…etc).	☐	☐
15. I know how such emergencies or disaster can affect the medication supply system (selection, quantification, procurement, storage, distribution).	☐	☐
16. I know the limits of my knowledge, skills, and readiness as a university/Medical student to act in disaster medicines situations, and I would know when I exceed them.	☐	☐
17. In case of the war, I know how to overcome the access to medicines problem to benefit my society.	☐	☐
18. I am familiar with the local emergency response system for medicines disasters.	☐	☐
19. I am familiar with the accepted process of 'examining problems in order to decide which ones are the most serious and must be dealt with first (triage principles)' used in disaster medicines situations.	☐	☐
20. I am familiar with the organizational logistics and roles among local and national agencies in disaster medicines response (i.e. taking decisions and measures) situations.	☐	☐
21. Realistic on-scene training is vital to an efficient and effective disaster medicines plan.	☐	☐
22. Disaster medicine is truly a systems-oriented specialty, and involved multiple responding agencies.	☐	☐

Part III (b): Attitude Assessment

Please indicate with a check mark (✓) your agreement or disagreement with the following statements.

	Strongly Agree	Agree	Neither Agree Nor Disagree	Disagree	Strongly Disagree
1. I consider myself prepared for the management of disasters medicines.	☐	☐	☐	☐	☐
2. I would feel confident in my abilities as a healthcare student in disaster medicines situation.	☐	☐	☐	☐	☐
3. I would be interested in educational classes on disaster medicines preparedness that relate specifically to the country situation	☐	☐	☐	☐	☐
4. I would be considered a key leadership figure in my community in a disaster medicines situation.	☐	☐	☐	☐	☐
5. I have personal/family emergency plans in place for disaster medicines situations.	☐	☐	☐	☐	☐
6. I have an agreement with loved ones and family members on how to execute our personal/family emergency and disaster medicines plans.	☐	☐	☐	☐	☐
7. I am able to describe my role in the response phase of a disaster medicines in the context of my college, the general public, media, and personal contacts.	☐	☐	☐	☐	☐
8. I would feel confident as a future manager or coordinator of a shelter/healthcare/ medication supply facility.	☐	☐	☐	☐	☐
9. I would be willing to be a future member of a healthcare facility/team in case of a medicines disaster.	☐	☐	☐	☐	☐
10. I feel reasonably confident I can care for patients independently without supervision of a physician in a medicines disaster situation.	☐	☐	☐	☐	☐
11. I would feel confident implementing emergency and disaster medicine plans and procedures.	☐	☐	☐	☐	☐

12. I would feel confident in providing medicine related education in case of disaster or emergency.	☐	☐	☐	☐	☐
13. As a University health or Medical student, I consider myself prepared for the management of medicines disasters.	☐	☐	☐	☐	☐
14. As a university health or medical student, I would feel confident in my abilities as a future healthcare provider and first responder in medicines disaster situation.	☐	☐	☐	☐	☐
15. There's enough awareness on "ways to stand wars and other humanity and natural emergencies among undergraduate students in University/Medical college	☐	☐	☐	☐	☐
16. I need more workshops and simulated training to be ready for dealing with disaster medicines.	☐	☐	☐	☐	☐

Part III (c): Readiness to Practice Assessment

Please indicate with a check mark (✓) your agreement or disagreement with the following statements.

	**Strongly Agree**	**Agree**	**Neither Agree Nor Disagree**	**Disagree**	**Strongly Disagree**	**Not Applicable**
1. My role in disaster medicines situations is clear.	☐	☐	☐	☐	☐	☐
2. I am ready to handle whatever potential risks emergencies exist in the community.	☐	☐	☐	☐	☐	☐
3. I am willing to attend the emergency medicine education incorporated in the undergraduate coursework.	☐	☐	☐	☐	☐	☐

4. I attended workshops/seminars about disaster medicine and it is enough for me to practice in real situation.	☐	☐	☐	☐	☐	☐
5. My undergraduate coursework enables me to be ready to practice in the settings of disaster (natural: eg- earthquakes and floods; or human made: eg- embargo or wars)	☐	☐	☐	☐	☐	☐
6. Other extracurricular resources (eg: internet, TV, radio and newspapers) enables me with a sufficient degree of readiness to practice under disaster.	☐	☐	☐	☐	☐	☐
7. I'm ready to practice under disaster knowing that some basic medications may not be available because of the disaster situation.	☐	☐	☐	☐	☐	☐
8. I need to be more trained on providing patient-centered care under the situation of disaster medicines.	☐	☐	☐	☐	☐	☐
9. The following are barriers that reduce my readiness to practice:	☐	☐	☐	☐	☐	☐

○ Lack of knowledge about medications disaster.◎ being unfamiliar with the new medications appearing during disasters. (The previous few questions are dealing with the same issue).	☐	☐	☐	☐	☐	☐
○ Disasters medicines are unlikely to occur in Pakistan.<br>○ It requires effort and time to be prepared.	☐	☐	☐	☐	☐	☐
